# Clinicopathologic Characteristics and Treatment Outcomes of Patients With Up-Front Metastatic Breast Cancer: Single-Center Experience in India

**DOI:** 10.1200/JGO.18.00265

**Published:** 2019-03-29

**Authors:** Ajay Gogia, Suryanarayana Vishnu S. Deo, Dayanand Sharma, Sanjay Thulkar, Rakesh Kumar, Prabhat S. Malik, Sandeep Mathur

**Affiliations:** ^1^ Institute of Rotary Cancer Hospital, All India Institute of Medical Science, New Delhi, India

## Abstract

**PURPOSE:**

Approximately 5% to 10% of patients with breast cancer present with up-front metastasis and carry a poor prognosis (5-year survival rates of approximately 20%). To date, little is known about the long-term outcome of patients with metastatic breast cancer from developing nations.

**MATERIALS AND METHODS:**

We performed an ambispective review of approximately 1,800 patients who were registered in breast cancer clinics between January 2012 and August 2018. Approximately 410 (22.8%) patients presented with up-front metastasis. Out of 410, 375 were considered for additional analysis. Clinical, pathologic, and radiologic details were obtained from the medical records.

**RESULTS:**

Median age of presentation was 49 years (range, 22 to 80 years), and median duration of symptoms was 6 months (interquartile range, 3-12 months). Baseline receptor status suggested that 234 patients (62.4%) were hormone receptor (HR) positive, 145 (38.6%) were human epidermal growth factor receptor positive, and 69 (18.6%) had triple-negative breast cancer. Various sites of metastasis were: visceral 219 (58.4%), bone only 100 (26.7%), nonregional lymph node metastasis 21 (5.6%), brain 10 (2.7%), and others 25 (5.8%). Approximately 309 patients (82.4%) received up-front chemotherapy, 192 HR-positive patients (82.1%) received endocrine therapy, and 78 human epidermal growth factor receptor–positive patients (53.8%) received targeted agents. Median progression-free survival was 14.2 months (95% CI, 12.7 to 16.8 months), and median overall survival (OS) was 31.7 months (95% CI, 25.8 to 38.2 months) for the cohort. Median time of follow-up was 22.2 months. On multivariable Cox regression analysis, HR-positive disease, good performance status (0 or 1), and oligometastasis were associated with better OS, whereas triple-negative breast cancer and liver and brain metastasis were associated with inferior OS.

**CONCLUSION:**

This is the first comprehensive study, to our knowledge, of metastatic breast cancer from India. HR-positive status, oligometastasis, and good performance status were associated with better outcomes.

## INTRODUCTION

Breast cancer is the most common cancer in Indian females (age-adjusted rate, 25.8 per 100,000 women; mortality rate, 12.7 per 100,000 women).^1^ It is hardly surprising that the majority of patients with breast cancer in India are still treated at locally advanced and metastatic stages.^2^ Approximately 5% to 10% of patients present with up-front metastasis, and 20% to 30% of patients develop metastasis during follow-up, as reported in the Western literature.^3^ Incidence of metastatic breast cancer (MBC) has been reported to be approximately 5% to 25% from various centers in India.^2,4^ MBC is unlikely to be cured; meaningful improvements in survival have been seen, coincident with the introduction of newer systemic therapies in Western literature.^5,6^ The median survival for MBC varies widely on the basis of subtype of tumor, sites of metastatic involvement, and burden of metastatic disease, and some patients experience long-term survival.^5,6^ MBC carries a poor prognosis in the Indian subcontinent; 5-year and 10-year overall survival have been reported to be 22% and 5%.^7^ The data are almost a decade old, and no new studies have been published from India regarding the impact of newer therapies on survival. As greater knowledge is brought forth regarding the specific molecular alterations associated with individual breast cancers, it will be of paramount importance to recognize prognostic factors and predictive factors that will help select specific therapy.

Advancement in the management of MBC has occurred recently, but there are many lacunae in the developing countries that prevent the achievement of maximum benefit for the patients (eg, tumor biology, poor socioeconomic status of patients, awareness of the patients regarding disease, availability of medications, nutritional status, hospital facility to provide regular care, and so on). With the knowledge of the existing literature, we designed an ambispective study to evaluate clinical and pathologic characteristics and treatment outcomes in the patients with up-front MBC.

## MATERIAL AND METHODS

### Study Design and Ethics

We designed an ambispective (both retrospective and prospective components) study of patients with up-front MBC who presented to our breast cancer clinic, which includes a dedicated team of medical oncology, surgical oncology, radiation oncology, pathology, radiology, and nuclear medicine departments. This study was conducted at All India Institute of Medical Science, New Delhi, India. In this study, we reviewed the records of patients who developed metastasis between January 2012 and August 2018. The Institute ethics committee provided clearance, and consent was obtained from patients, who were recruited prospectively.

**FIG 1:**
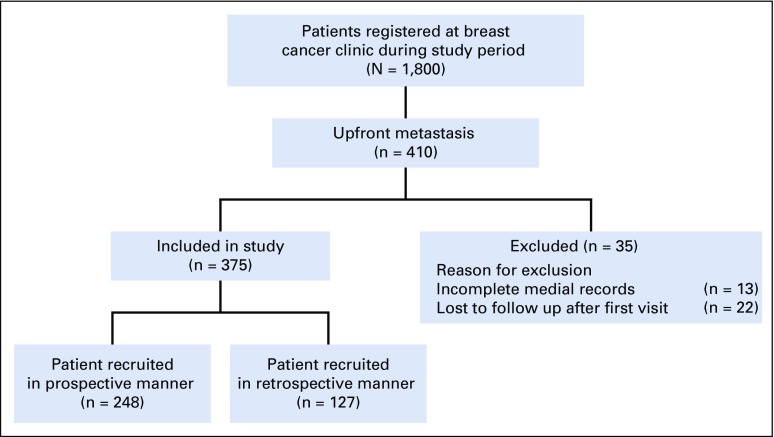
CONSORT diagram. Flow diagram showing total patients with metastatic breast cancer screened, patients included prospectively and retrospectively, and reasons for exclusion.

During the study period, approximately 1,800 patients were registered in the breast cancer clinic; 410 patients (22.8%) presented with up-front metastasis. Out of 410 patients, 375 patients were considered for analysis (248 recruited prospectively and 127 retrospectively), and 35 patients were excluded (13 because of incomplete medical records and 22 because of loss to follow-up after first visit; Fig 1). Data were retrieved from medical records regarding clinical presentation, radiographic features, molecular biomarkers (hormone receptor [HR; estrogen receptor (ER) and progesterone receptor (PR)] and human epidermal growth factor receptor 2 [HER2/*neu*]), treatment schedules, and survival.

### Treatment Protocol

Institute protocol for up-front MBC management is single agent or combination chemotherapy, endocrine therapy (tamoxifen or aromatase inhibitor), and targeted therapy (trastuzumab or pertuzumab) individualized as per receptor status. Combination chemotherapy was given to patients with oligometastasis. Protocol for combination chemotherapy is anthracycline plus taxane or taxane plus platinum-based chemotherapy (four cycles of fluorouracil 600 mg/m^2^ plus epirubicin 75 mg/m^2^ plus cyclophosphamide 600 mg/m^2^ followed by four cycles of docetaxel 85 mg/m^2^ or docetaxel 75 mg/m^2^ plus carboplatin, area under the curve, 6) once in 3 weeks. Patients who did not have oligometastatic disease received single-agent chemotherapy with taxanes (docetaxel 85 mg/m^2^ once in 3 weeks or paclitaxel 80 mg/m^2^ once per week).

### Pathologic Assessment

Hormone and HER2/*neu* were tested by the standard immunohistochemical (IHC) method. Allred scoring was used for reporting ER/PR receptor status. The percentage of cells showing ER positivity and intensity was recorded. A score of 3 or more was considered positive.^8^ IHC testing to determine ER, PR, and HER2/*neu* status was performed using the standard procedures on 4-μm sections of paraffin-embedded tissue specimens stained with the monoclonal antibodies (1:400; Thermo Scientific, Waltham, MA; 1:400; abcam, Cambridge, MA; and 1:100, Thermo for ER, PR, and HER2/*neu*, respectively). Nuclear staining greater than 1% of tumor cell was considered as positive for ER and PR. Patients were considered HER2 positive if they had IHC 3+-positive or fluorescence in situ hybridization was amplified (more than six copies of HER2/*neu* gene or HER2/*neu*:centromere enumerator probe 17 ratio of more than two). HER2/*neu* status was tested as per the ASCO/College of American Pathologists guidelines.^9^ A score of 3+ was considered positive, and 2+ was considered equivocal. All 2+ results of HER2/*neu* were confirmed by the fluorescence in situ hybridization method as per standard guidelines. Histologic type was assessed according to the World Health Organization standards.

### Response Evaluation

All patients underwent evaluation with complete clinical history, physical examination, and radiologic evaluations (bone scintigraphy, computed tomography scan, magnetic resonance imaging, positron emission tomography). Treatment response was monitored by clinical examination at every visit. Radiologic response to treatment was assessed by the Response Evaluation Criteria in Solid Tumors (RECIST) version 1.1.^10^ Response at 6 months of therapy was documented for partial response, complete response, or stable disease.

### Outcome Variables

Data regarding patient’s age, sex, menopausal status, performance status (PS), site of metastasis, number of metastases, histology, receptor status (ER, PR, and HER2 receptors), response assessment, and survival were recorded from medical records. Bone only, lung, liver, brain, and nonregional lymph nodes were recorded for site of metastasis. Lung and liver metastases were analyzed irrespective of bone and lymph node metastases, and brain metastasis was analyzed irrespective of any other site of metastasis.

### Statistical Analysis

Nominal data were presented as number and percentage, and continuous data were presented as median and range. Median duration of follow-up was calculated by reverse Kaplan-Meier method. Overall survival (OS) was defined as the duration from the start of treatment to last visit or death. Progression-free survival (PFS) was calculated from the date of start of treatment to the first date of documented progressive disease or the date of death as a result of breast cancer progression. The Kaplan-Meier method was used for survival analysis. Estimated survival data are presented as median with 95% CI and proportions. Univariable analysis was performed by Cox regression for estimated OS; the following variables were included: age (≤ 50 or > 50 years), receptor status, site of metastasis, and oligometastasis (five or fewer metastases involving one or two organs). Multivariable analysis was performed for those parameters that were significant in the univariable analysis by Cox regression. Results of univariable and multivariable analyses are presented in *P* value and hazard ratio (95% CI). Differences were considered statistically significant for *P* values < .05. Patients were contacted by telephone for present status, and survival data were censored on November 30, 2018. Data were analyzed in Stata 13 and SPSS software (College Station, TX).

## RESULTS

### Baseline Characteristics

Median age of presentation was 49 years (range, 22 to 80 years; Table 1). Median duration of symptoms was 6 months (interquartile range, 3-12 months) in patients who presented with up-front MBC. Right side breast cancer was present in 173 (46.1%), left side in 184 (49.1%), and bilateral in 18 (4.8%) patients. Eastern Cooperative Oncology Group (ECOG) PS was 0, 1, 2, 3, and 4 in 48 (12.8%), 241 (64.3%), 58 (15.5%), 11 (2.9%), and 17 (4.5%) patients, respectively, at presentation.

**TABLE 1:**
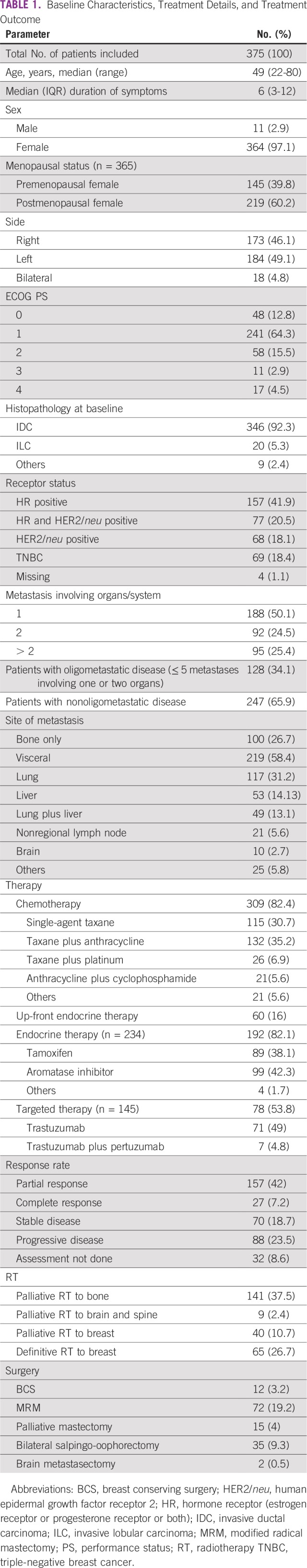
Baseline Characteristics, Treatment Details, and Treatment Outcome

Metastasis involving a single organ was present in 188 (50.1%), two organs in 92 (24.5%), and more than two organs in 95 (25.4%) patients. The most common site of metastasis was visceral metastasis (219 [58.4%] lung, liver, and both [lung plus liver] in 117 [31.2%], 53 [14.13%], and 49 [13.1%], respectively), followed by bone-only metastasis in 100 (26.7%), nonregional lymph node in 21 (5.6%), brain in 10 (2.7%), and other site of metastasis in 25 (5.8%) patients. The most common histology was infiltrating ductal carcinoma in 346 patients (92.3%) followed by infiltrating lobular carcinoma in 20 patients (5.3%). HRs (ER or PR or both) were positive in 157 patients (41.9%). HR and HER2/*neu* were positive in 77 patients (20.5%). Only HER2/*neu* was positive in 68 patients (18.1%), 69 patients (18.4%) were triple negative, and data were missing in four patients (1.1%). Approximately 128 patients (34.1%) had oligometastatic disease (five or fewer metastases involving one or two organs), and the rest did not meet the criteria for oligometastasis.

### Treatment Details and Outcome

Out of 375 patients, in first-line therapy 115 patients (30.7%) received single-agent chemotherapy, 179 patients (47.7%) received combination chemotherapy, and 60 patients (16%) received up-front endocrine therapy (Table 1). Out of 234 patients who were positive for HR, 192 (82.1%) received endocrine therapy (89 [38.1%] tamoxifen, 99 [42.3%] aromatase inhibitor, and four [1.7%] others). Out of 145 patients who were positive for HER2/*neu*, 78 patients (53.8%) received targeted therapy (71 [49%] trastuzumab, seven [4.8%] trastuzumab plus pertuzumab).

Palliative radiotherapy was given to 141 patients (37.5%) for bone metastasis, nine patients (2.4%) for brain and spine metastasis, and 40 patients (10.7%) for breast. Approximately 65 patients (26.7%) received definitive locoregional radiotherapy. Approximately 72 patients (19.2%) underwent modified radical mastectomy, 12 patients (3.2) had breast-conserving surgery, 15 patients (4%) had palliative mastectomy, 35 patients (9.3%) had salpingo-oophorectomy, and two patients (0.5%) had brain metastasectomy. Overall response was present in 184 patients (49.1%; partial response, 157 [42%] and complete response 27 [7.2%]), stable disease in 70 patients (18.7%), and 88 patients (23.5%) had progressive disease. Response assessment was not done in 32 patients (8.6%).

### Survival Analysis

**FIG 2:**
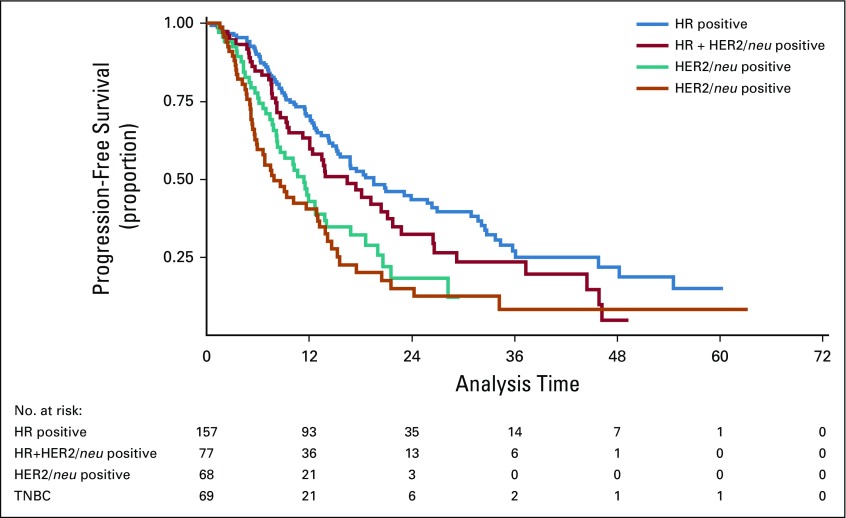
Kaplan-Meier estimate of progression-free survival of various receptor subtypes. HR, hormone receptor; HER2/*neu*, human epidermal growth factor receptor 2; TNBC, triple-negative breast cancer.

**FIG 3:**
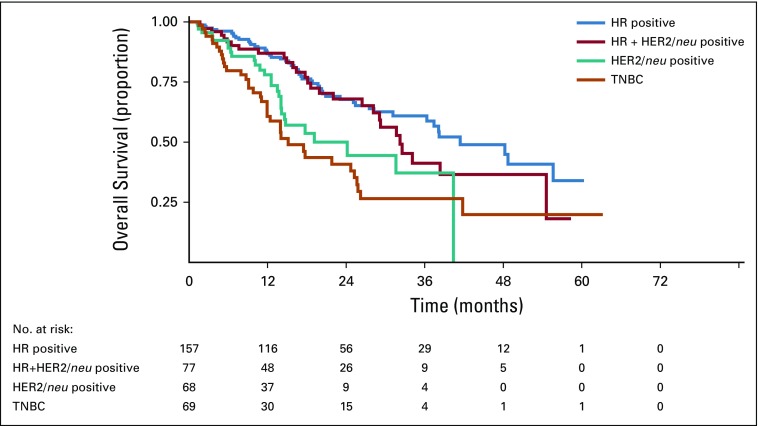
Kaplan-Meier estimate of overall survival of various receptor subtypes. HR, hormone receptor; HER2/*neu*, human epidermal growth factor receptor 2; TNBC, triple-negative breast cancer.

Median time of follow-up was 22.2 months (95% CI, 19.3 to 26 months). Median PFS for the cohort was 14.2 months (95% CI, 12.7 to 16.8 months), and median OS was 31.7 months (95% CI, 25.8 to 38.2 months). Median PFS was 19.5 months (95% CI, 15.4 to 26.9 months), 16.4 months (95% CI, 12 to 21.7 months), 11.4 months (95% CI, 8.2 to 13.8 months), and 7.9 months (95% CI, 5.7 to 12.8 months) for HR, HR plus HER2/*neu*, HER2/*neu*, and triple-negative breast cancer (TNBC) patients, respectively (Fig 2). Median OS was 41.4 (95% CI, 30 to 52.8), 32.2 (95% CI, 27 to 37.4), 24.1(95% CI, 11.3 to 37), 17.5(95% CI, 11 to 19.2) for HR, HR plus HER2/*neu*, HER2/*neu*, and TNBC (Fig 3). Median PFS of bone-only metastasis was 23.9 months (95% CI, 19.5 to 32.5 months), visceral metastasis 14.2 months (95% CI, 12.7 to 16.8 months), nonregional lymph node metastasis 20.6 months (95% CI, 5.3 to 30.9 months), and brain metastasis 6.7 months (95% CI, 1.1 to 12.9 months). Median OS of bone-only metastasis was 48.2 months (95% CI, 36 to 60.6 months), visceral metastasis 21.9 months (95% CI, 16.1 to 27.9 months), nonregional lymph node metastasis 38.2 months (95% CI, 21.3 to 55.1 months), and brain metastasis 14.6 months (95% CI, 8.9 to 20.2 months). Median PFS of patients with good ECOG PS (0 and 1) was 15.6 months (95% CI, 13.8 to 19.5 months); median PFS of patients with poor ECOG PS^2-4^ was 11.6 months (95% CI, 9.1 to 13.2 months). Median OS of patients with good ECOG PS was 38.2 months (95% CI, 27.2 to 49.2 months); median OS of patients with poor ECOG PS was 14.7 months (95% CI, 11.4 to 18.1 months).

Results of univariable and multivariable analyses are listed in Table 2. On univariable analysis, HR-positive disease, bone-only metastasis, ECOG PS 0 or 1, and oligometastasis were associated with statistically better OS (*P* values of .01, .01, .01, and .01 and hazard ratios [95% CIs] of 0.54 [0.39 to 0.76], 0.41 [0.26 to 0.63], 0.36 [0.25 to 0.52], and 0.24 [0.15 to 0.38], respectively). TNBC, lung metastasis, liver metastasis, and brain metastasis were associated with statistically inferior survival (*P* values of .01, .01, .01, and .01 and hazard ratios [95% CIs] 2.24 [1.53 to 3.26], 2 [1.44 to 2.79], 2.31 [1.64 to 3.24], and 2.86 [1.33 to 6.16], respectively; Table 2). On multivariable analysis, HR-positive disease, ECOG PS 0 or 1, and oligometastasis were associated with statistically better survival (*P* values of .01, .01, and .01; hazard ratios [95% CIs] were 0.56 [0.35 to 0.89], 0.45 [0.31 to 0.65], 0.24 [0.15 to 0.40]), and liver metastasis, brain metastasis, and TNBC were associated with statistically poor survival (*P* values of .01, .04, .01 and hazard ratios [95% CIs] of 1.94 [1.32 to 2.83], 2.22 [1.1 to 4.84], and 2.19 [1.31 to 3.67], respectively; Table 2).

**TABLE 2:**
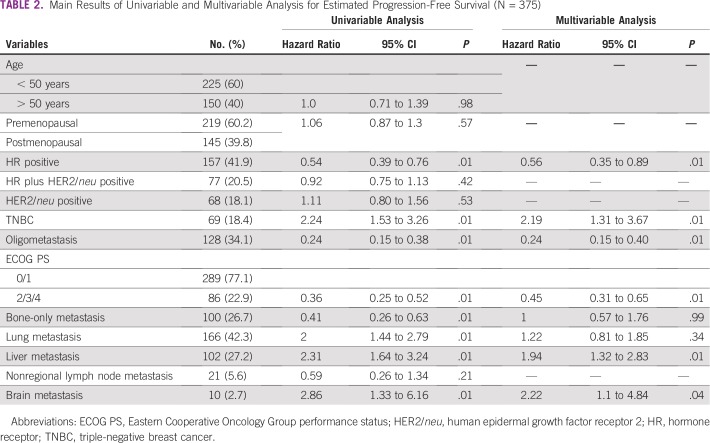
Main Results of Univariable and Multivariable Analysis for Estimated Progression-Free Survival (N = 375)

In a subset analysis of HER2/*neu*-positive patients, those who received targeted therapy showed better PFS and OS than those who did not. Median PFS of patients who did not receive targeted therapy was 9.3 months (7.6 to 13.5 months), whereas median PFS of patients who received targeted therapy was 19.7 months (12.4 to 21.7 months). Median OS of patients without targeted therapy was 24.1 months (14.5 to 32.2 months), whereas median OS of patients who received targeted therapy was not reached. Expected OS at 2 and 4 years was 51.2% and 9.7% for patients who did not receive targeted agents and 68% and 58.2% for those who received targeted agents. *P* value was .01, and hazard ratio (95% CI) was 0.4 (0.22 to 0.7).

## DISCUSSION

Breast cancer is the most common cancer in women worldwide, with widely variable incidence among countries and regions. As per the Indian Council of Medical Research Population-based Cancer Registry data, breast cancer is the most common cancer among women in urban registries of Delhi, Mumbai, Ahmedabad, Calcutta, and Trivandrum, where it constitutes more than 30% of all cancers in females.^11^ In the rural Population-based Cancer Registry of Barshi, breast cancer is the second most common cancer in women after cancer of the uterine cervix.^11^

We have compared this study with various previous studies published in the Western literature, as listed in Table 3. In general, breast cancer has been reported to occur a decade earlier in Indian patients compared with their Western counterparts. Although the majority of patients with breast cancer in Western countries are postmenopausal and in their 60s and 70s, the picture is quite different in India, with premenopausal patients constituting approximately 50% of all patients.^2^ More than 80% of Indian patients are younger than 60 years of age. The average age of patients with breast cancer has been reported to be 50 to 53 years in various population-based studies done in different parts of the country.^12^ In the present study we have documented a median age of 48 years (range, 22 to 80 years). It is hard to find data on a nonselected population of patients with MBC. In previous studies from Western countries, the median age of presentation was 55 to 60 years (Table 3). In a recent study from various races and ethnicities in the US population by Iqbal et al,^13^ the median age of presentation for all stages was 55 to 60 years. The present study documented that approximately 39.8% of patients were premenopausal and 60.2% were postmenopausal, whereas studies from the Western world documented 70% to 80% postmenopausal patients (Table 3).

**TABLE 3:**
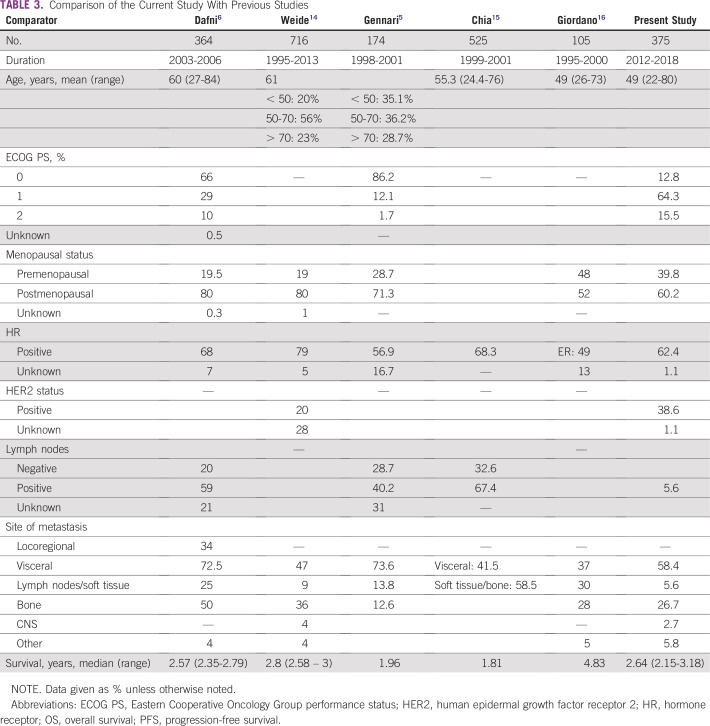
Comparison of the Current Study With Previous Studies

It is hardly surprising that the majority of patients with breast cancer in India are still treated at locally advanced and metastatic stages.^2,4^ Approximately 6% to 25% of patients were documented with metastasis at baseline in five major centers in India.^2^ In patients who had metastasis in this study, 22% presented with up-front metastasis. Single-center studies usually have referral bias, but there are no population data available for various stages of breast cancer from India. If we compare data with Western literature, Iqbal et al^13^ documented 3% to 8% of patients present in stage IV at baseline in various races and ethnicities in the US population.^13^

Common sites of metastasis are bone, lung, liver, lymph nodes, chest wall, and brain.^5^ HR-positive tumors spread to the bone. HR-negative and/or HER2 tumors are likely to spread to the viscera; however, this is not a rule of thumb. Lobular carcinoma (as opposed to ductal) is associated with serosal metastases (pleura and abdomen).^6^ These may be symptomatic or asymptomatic and detected at initial presentation or during follow-up. The goal of managing MBC is to allow the patient to regain and maintain the best possible quality of life and to prolong her life to the extent possible. A multidisciplinary approach to breast cancer treatment that is so vital is available only at a few select regional centers in India. The data available on various issues relating MBC care in India are scant and heterogeneous. At baseline, approximately 77% of patients were of ECOG PS 0 and 1, which is 10% to 20% lower than in Western literature (Table 3). A limitation in our study is that we were not able to include patients with advanced-stage breast cancer who directly presented in the emergency department and succumbed to disease, as these patients were not registered in breast cancer clinic. In this study, HR was positive in 62.4% of patients; previous studies documented approximately 55% to 80% (Table 3). Survival of patients with MBC is improving because of the availability of newer therapies; it has been well documented in previous studies.^7,16^ Median OS with aromatase inhibitor has been documented at 30 to 33 months.^17^ Combined treatment with ovarian suppression and tamoxifen was superior to treatment with ovarian suppression or tamoxifen alone, with median overall survival of 3.7, 2.5, and 2.9 years.^18^ In this study of an unselected population, where most of HR-positive patients received chemotherapy followed by endocrine therapy, OS was 41.4 months. Median PFS and OS for HER2/*neu*-positive breast cancer in patients receiving trastuzumab, pertuzumab, and taxane versus trastuzumab and taxane was 18.5 versus 12.4 months and 56.5 versus 40.8 months, respectively.^19,20^ In a subset analysis of HER2/*neu*-positive patients, those who received targeted therapy showed better PFS and OS. Median PFS of patients who received targeted agents was 19.7 months, and median OS was not reached. Patients receiving anthracycline and taxane-based chemotherapy showed PFS of 6 to 7 months and OS of 18 to 20 months in the first-line setting.^21-24^ In our study, patients who were HER2/*neu* positive who received chemotherapy showed a PFS of 9.7 months and OS of 24.1 months, whereas patients with TNBC who received chemotherapy had a median PFS of 7.1 months and OS of 17.5 months. In this study, HR-positive status, oligometastasis, and good PS were associated with significantly better outcomes, whereas liver and brain metastasis and TNBC were associated with significantly worse outcome. These results corroborated with most previous studies of MBC.^6,10,16^

MBC is treated by a multidisciplinary treatment approach. This study provides significant evidence of improvement in the prognosis of patients with MBC within the last 15 years, while taking into account the beneficial effect of all significant prognostic factors (good PS, positive HR status, absence of visceral metastasis at entry, and fewer metastatic sites). In addition, there is considerable evidence strongly suggesting that this improvement can be attributed to the use of new systemic therapy agents in the management of the disease.
